# Developing an artificial intelligence tool for detecting fractures of child abuse: preliminary findings

**DOI:** 10.1007/s00330-026-12513-8

**Published:** 2026-04-04

**Authors:** Samuel Evans, Nihal Chanian, Esther Bezzina, Amaka C. Offiah

**Affiliations:** 1 University of Sheffield Medical School, University of Sheffield, Sheffield, UK; 2 Sheffield Children’s NHS Foundation Trust, University of Sheffield, Sheffield, UK; 3 Division of Clinical Medicine, University of Sheffield, University of Sheffield, Sheffield, UK

**Keywords:** Artificial intelligence, Child, Deep learning, Fractures, Bone, Physical abuse

## Abstract

**Objectives:**

Approximately 6.9% of children in the United Kingdom have suffered physical abuse. Fractures are a common sign and must not be overlooked due to high recurrence and mortality rates. We aimed to train and assess the diagnostic accuracy of a deep learning-based artificial intelligence model (BoneView) in detecting inflicted fractures.

**Materials and methods:**

This pragmatic retrospective diagnostic accuracy pilot study focuses on children under 5 years old who underwent skeletal survey examinations for suspected physical abuse at a single tertiary centre between 1st January 2000 and 31st December 2023. Radiographs were extracted from the Picture Archiving and Communication System and divided to retrain and test the model. Radiology reports and retrospective review by one observer were used as the reference standard.

**Results:**

Our total dataset included 1740 patients (mean age, 8.77 months ± 8.343 [standard deviation], 1026 males). The model’s baseline performance recorded an area under the receiver operating curve (AUC) of 0.46 (95% CI: 0.38, 0.57), with a sensitivity of 44% (95% CI: 35%, 58%) and a specificity of 61% (95% CI: 52%, 71%). For preliminary model training, 329 of 1227 positive studies were annotated, yielding a revised AUC of 0.55 (95% CI: 0.48, 0.66), sensitivity of 52% (95% CI: 43%, 64%), and specificity of 67% (95% CI: 58%, 78%).

**Conclusion:**

Preliminary training of a novel AI tool for detecting inflicted fractures yielded improved results from baseline performance. This justifies the completion of annotation and further training of this AI tool to potentially achieve clinically acceptable performance.

**Key Points:**

***Question*** *Double reporting of skeletal surveys is vital for identifying fractures caused by physical abuse, but some departments lack the expertise to double report these investigations*.

***Findings*** *Preliminary retraining of a commercially available deep learning algorithm using radiographic skeletal surveys led to improved inflicted fracture detection accuracy*.

***Clinical relevance*** *Training this deep learning algorithm using relevant imaging enhances its performance. An accurate tool for automated skeletal survey interpretation may improve outcomes for physically abused children by offering an additional diagnostic opinion*.

**Graphical Abstract:**

**Figure:**
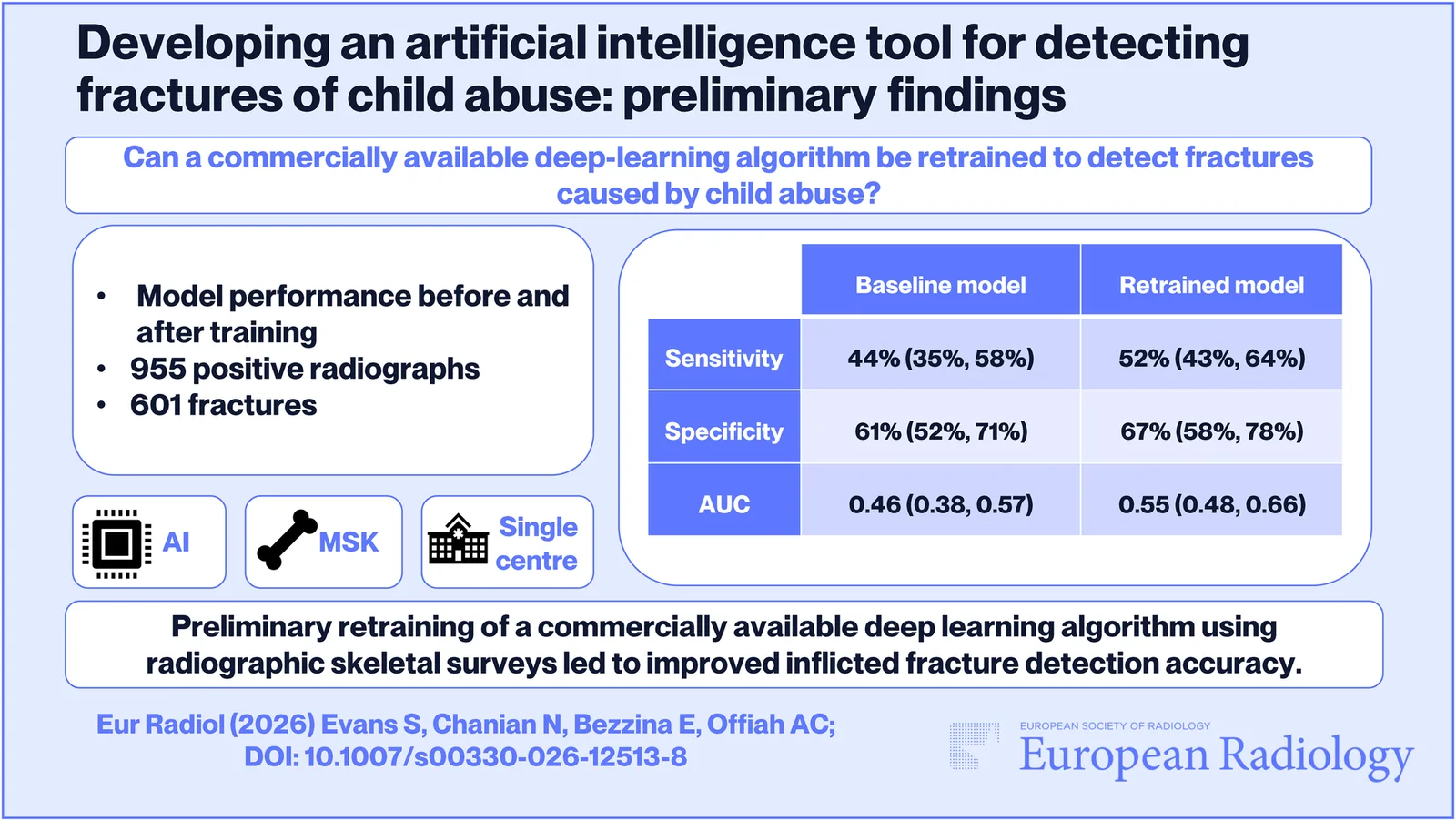

## Introduction

Physical abuse affects approximately 6.9% of children in the United Kingdom (UK) [1]. Fractures are a common feature, occurring in up to 55% of physically abused children [2]. Timely recognition of abuse-related fractures is vital as children left unprotected face a high risk of recurrence (15–50%) and a mortality rate of up to 10% [3, 4].

While skeletal surveys are the first line of investigation in suspected physical abuse (SPA), fractures typically associated with abuse, such as classic metaphyseal and rib fractures, can be particularly challenging to identify radiographically, especially when acute or non-displaced [5, 6].

To minimise the possibility of oversight, the Royal College of Radiologists recommend that skeletal surveys be reviewed within 24 h by two radiologists with at least 6 months of specialist paediatric radiology training, including experience with SPA [5]. However, a UK survey revealed that only 52% of departments have a paediatric radiologist, and an audit at a tertiary children’s centre in the UK found that only 75% of skeletal surveys were reported within the recommended 24-h timeframe [7]. An artificial intelligence (AI) tool could fill this gap by acting as the first reviewer.

In recent years, AI has demonstrated promising results within the field of medical imaging, with 79% of healthcare-related AI products cleared in 2023, focusing on radiology applications [8]. In March 2023, Gleamer’s BoneView AI software tool became the first to receive Food and Drug Administration clearance for detecting appendicular fractures on radiographs in children over two years old [9]. However, children under the age of one are most vulnerable to abusive fractures, which can occur at multiple sites, not just the appendages [10].

This study aims to externally validate the deep learning-based AI model BoneView for detecting inflicted fractures in children and subsequently retrain a model inspired by the latest iteration (updated mid-April 2024) using a preliminary dataset. Model performance following retraining will be descriptively compared with baseline performance using standard measures of diagnostic accuracy. This will provide insight into the feasibility of developing a reliable model for detecting abuse-related fractures. No large-scale studies have investigated this topic, though previous research using deep learning models has shown encouraging results in paediatric diaphyseal fracture detection [11–13].

## Materials and methods

### Initial evaluation of BoneView for the detection of inflicted fractures

This single-centre pragmatic retrospective pilot study received Health Research Authority (HRA) and Local Research and Innovation (R&I) approval. The need for patient consent was waived. BoneView’s initial performance was independently ascertained by conducting a retrospective diagnostic accuracy study prior to Gleamer’s involvement in post-training analysis. Initial performance was expected to be low as Gleamer’s current dataset does not contain significant numbers of images of children under 2-years-old.

Sixteen skeletal surveys (367 images) from 16 patients were selected from our hospital’s Picture Archiving and Communication System (PACS), performed between 01/01/2011 and 31/12/2021, by A.C.O. (a consultant paediatric musculoskeletal radiologist with 20 years of experience) to include a mixture of fracture types and locations. Radiographs were only included when A.C.O. agreed with the clinical (double) report provided by the radiologists and/or when follow-up imaging confirmed the presence of injury. This was treated as the ground truth. Other forms of imaging (e.g., CT, MRI and ultrasound) were excluded, and frontal chest, nasal bones, skull, abdomen and lateral whole-body films were omitted from the analysis due to the software, which was trained using radiographs of the appendicular skeleton, being unable to recognise them.

Radiographs were anonymised and uploaded to BoneView for analysis. AI predictions were compared to the ground truth. Sensitivity and false negative rate were calculated at the lesion level. Raw data were analysed using the IBM SPSS Statistics package (version 29.0.1.0). Sensitivity is represented by a positive diagnostic test and positive ground truth. Where the AI output was ‘doubt’, this was considered a ‘positive’ prediction, and the expert report was consulted to categorise the result as true or false positive. False positives included any abnormality highlighted by the software which was not in the area of interest defined by the ground truth. The workflow process can be visualised in Supplemental Fig. 1.

### Retraining of BoneView

#### Dataset collection

An advanced search was conducted using our hospital’s PACS. Due to the absence of specific guidelines on sample size for model training, we included consecutive patients under 5 years old who underwent a skeletal survey for SPA between 01/01/2010 and 31/12/2023. Both initial and follow-up skeletal surveys, as well as trauma-related radiographs prompting or occurring during SPA investigations, were included. Studies were excluded if image quality was significantly degraded by artefact, if genetic conditions affecting skeletal morphology (e.g., osteogenesis imperfecta or other skeletal dysplasia) were present, if reports were inconclusive even after review by A.C.O., or if the studies were mislabelled or duplicates. All relevant studies were then transferred to Gleamer’s online software annotation tool.

### Ground truth

The ground truth was defined according to the skeletal survey reports. These are made by at least one consultant paediatric radiologist. Since 2017, all skeletal surveys have been double-reported by two consultant paediatric radiologists. Inconclusive reports (*n* = 164) were reviewed by A.C.O., and studies were excluded if doubt remained. For images without an available report in PACS (*n* = 400), where A.C.O. was able to provide a definitive opinion, this was used as the ground truth.

### Annotation

Before annotation, S.E. (a third-year medical student) and A.C.O. partook in a 1-h training session to learn how to use Gleamer’s online annotation tool. Fractures were then annotated by S.E. using bounding boxes. The site, type, displacement, and age of each fracture and the presence of external hardware were also recorded. All S.E.’s annotations were validated by A.C.O.

### Data partition

The annotated radiographs and an equal number of negative studies were added to Gleamer’s existing dataset. The data were partitioned into training and test sets in an 8:2 ratio at the study level.

### Model training

No data pre-processing was required for this study. The retrained model is based on a proprietary deep-learning architecture for object detection, inspired by Gleamer’s BoneView. Note that the baseline and retrained models do not represent the actual performance of BoneView since BoneView is composed of several models, whereas this study only covers one. Supervised learning was employed, and since the model was not learning from scratch, transfer learning was also employed [14].

### Statistical methods

Data were recorded in a Google Sheets spreadsheet, and dataset characteristics were analysed using SPSS IBM Statistics. For model performance, statistical analysis was conducted by Gleamer, as raw data were not shared for confidentiality reasons.

Study-wise model performance was calculated using the following definitions:

- True positive = The study is positive, and the AI tool identifies all fractures with bounding boxes
- True negative = The study is negative, and the AI tool outputs no bounding boxes
- False positive = The study is negative, but the AI tool outputs at least one bounding box
- False negative = The study is positive, but at least one fracture is missed by the AI tool

Sensitivity and specificity were calculated at an operating point of 0.5 false positives per study.

## Results

### Initial evaluation of BoneView for the detection of inflicted fractures

Sixteen patients were included (median age, 2.4 months, interquartile range: 2.14, 9 males). Of the 367 images, 306 were interpreted by the software. A total of 75 fractures were present in the dataset. Sensitivity was calculated as 46.1% (95% confidence interval [CI]: 35.6%, 56.4%). Supplemental Table 1 shows the breakdown of the data per anatomical site. Fractures were classified as acute (*n* = 18) and healing (*n* = 57), where sensitivity was 50% (95% CI: 28.1, 71.9%) and 47.4% (95% CI: 34.7, 60.2%), respectively. This is shown in Supplemental Table 2. Data were also broken down into fracture types, including shaft (*n* = 14), metadiaphyseal (*n* = 8), supracondylar (*n* = 5) and classic metaphyseal fractures (*n* = 28). Of the 28 classic metaphyseal fractures, eight were correctly identified, giving a sensitivity of 28.6%. The breakdown of this data is shown in Supplemental Table 3.

### Retraining of BoneView

#### Dataset collection and partition

**Fig. 1:**
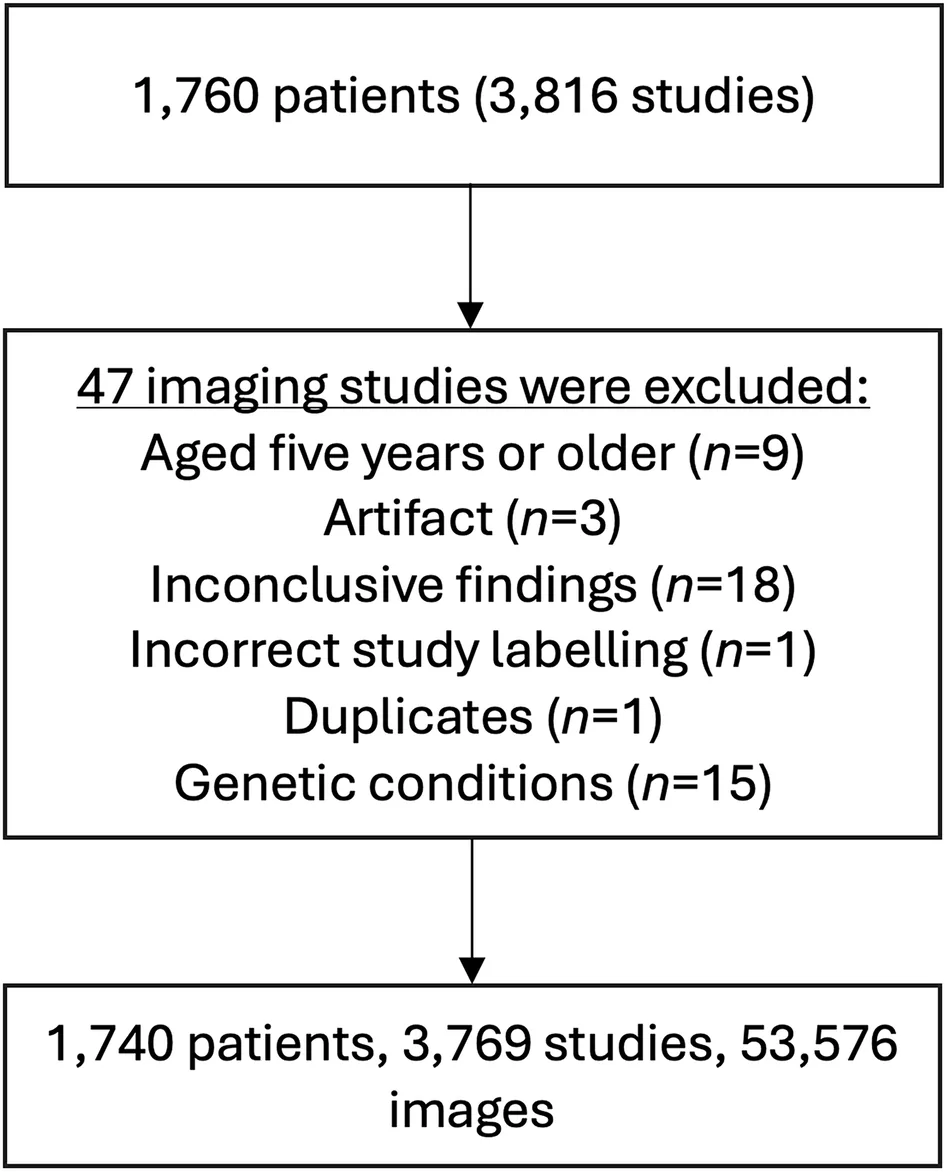
Flowchart showing patient selection

A total of 3816 radiographic studies from 1760 patients were acquired. Forty-seven studies were excluded, leaving 3769 studies from 1740 patients. The reasons for exclusions are shown in Fig. 1. Annotation was completed for 329 of 1227 positive studies, constituting 955 positive radiographs and 601 fractures (as multiple views can capture the same fracture). Table 1 shows the data partition into training and test sets.

**Table 1:** Distribution of data into training and test sets

	Number of studies	Number of images	Number of fractures
Training set	526	7954	463
Test set	132	1841	138
Total	658	9795	601

### Population characteristics

Of 1740 patients in our total dataset, 59.5% were male, with the sex of five patients unknown. The median age was 6 months (interquartile range: 11), with the youngest patient being 0 days old and the oldest 4 years old.

### Model performance for all fractures

**Fig. 2:**
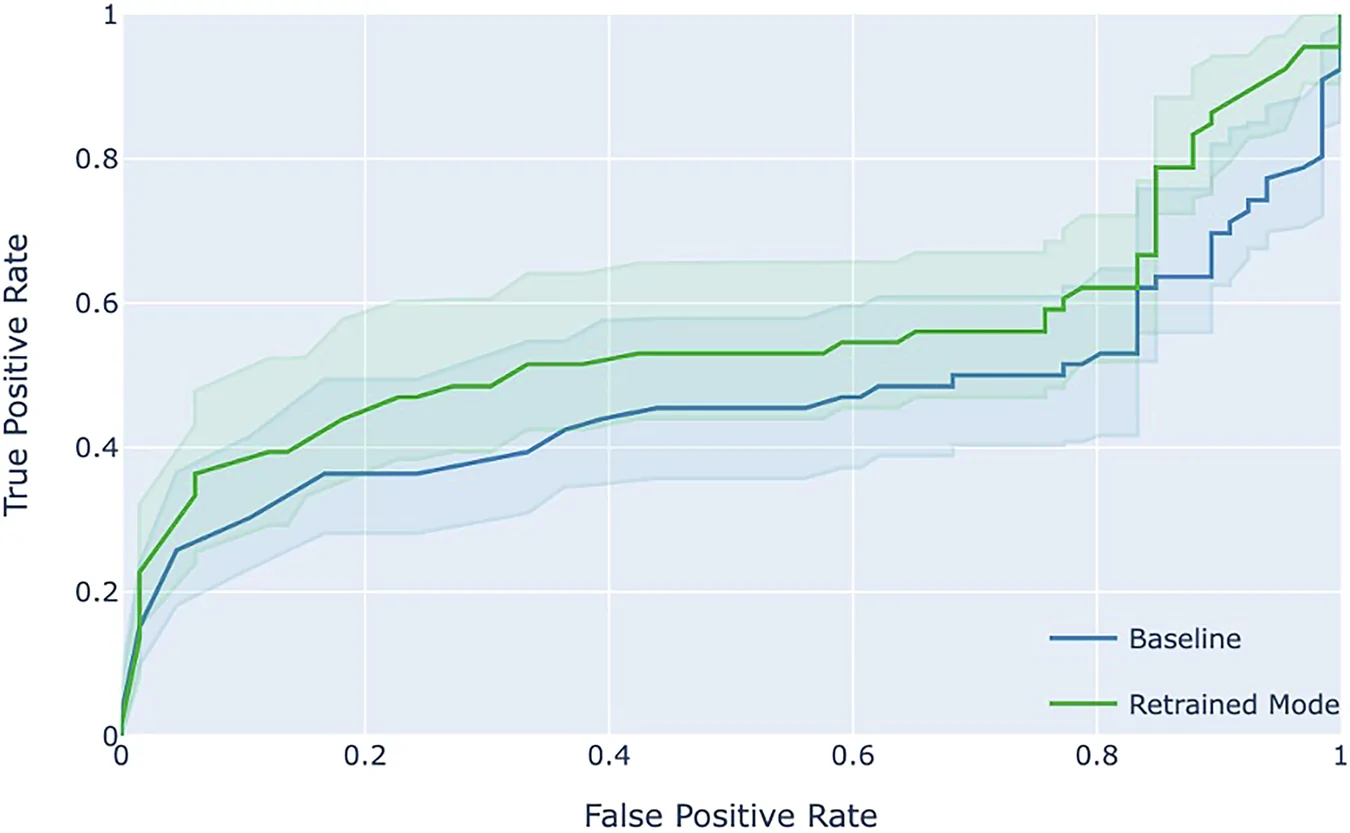
ROC curve for baseline and retrained models. Note: the blue line represents the ROC curve for the baseline model, and the green line shows the ROC curve for the retrained model. The blue and green shaded areas represent 95% confidence intervals for the baseline and retrained model, respectively. Graph courtesy of Gleamer

**Fig. 3:**
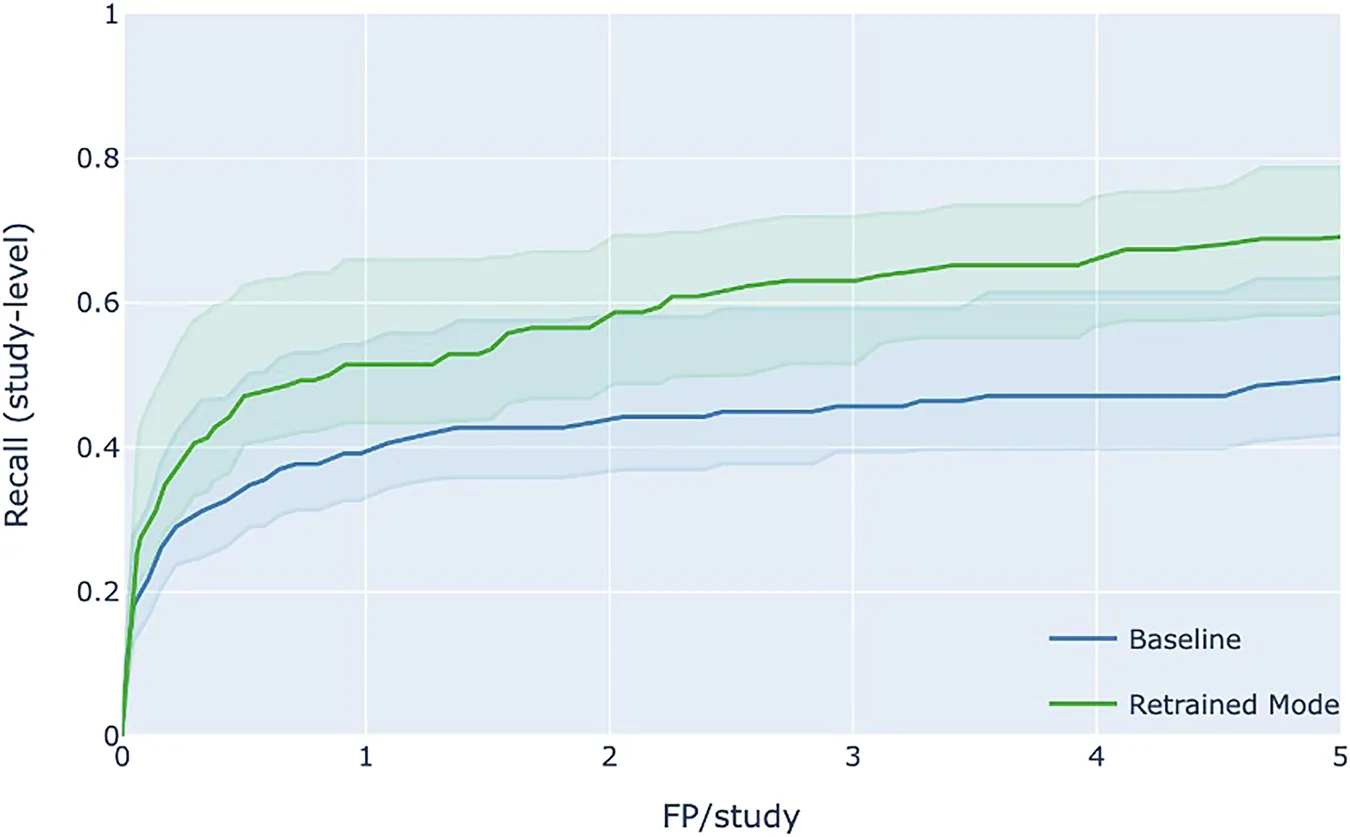
FROC curve for baseline and retrained models. Note: The blue line represents the FROC curve for the baseline model, and the green line shows the FROC curve for the retrained model. The blue and green shaded areas represent 95% confidence intervals for the baseline and retrained model, respectively. Graph courtesy of Gleamer

The baseline model achieved an area under the receiver operating curve (AUC) of 0.46 (95% CI: 0.38, 0.57), with a study-wise sensitivity of 44% (95% CI: 35%, 58%) and a specificity of 61% (95% CI: 52%, 71%). After retraining, the model’s AUC improved to 0.55 (95% CI: 0.48, 0.66), along with increases in sensitivity to 52% (95% CI: 43%, 64%) and specificity to 67% (95% CI: 58%, 78%) (Table 2). The receiver operating characteristic (ROC) and free-response receiver operating characteristic (FROC) curves are shown in Figs. 2 and 3, respectively, for the baseline and retrained models.

**Table 2:** Performance metrics for the baseline and retrained models

	Baseline model	Retrained model
Sensitivity	44% (35%, 58%)	52% (43%, 64%)
Specificity	61% (52%, 71%)	67% (58%, 78%)
AUC	0.46 (0.38, 0.57)	0.55 (0.48, 0.66)

Note: Sensitivity and specificity are calculated at an operating point of 0.5 false positives per study. Evaluation metrics are calculated at the study level, derived from the ROC curve. Values in parentheses are 95% confidence intervals, computed via bootstrapping

*AUC* area under the receiver operating characteristic curve

### Model performance for rib fracture detection

**Fig. 4:**
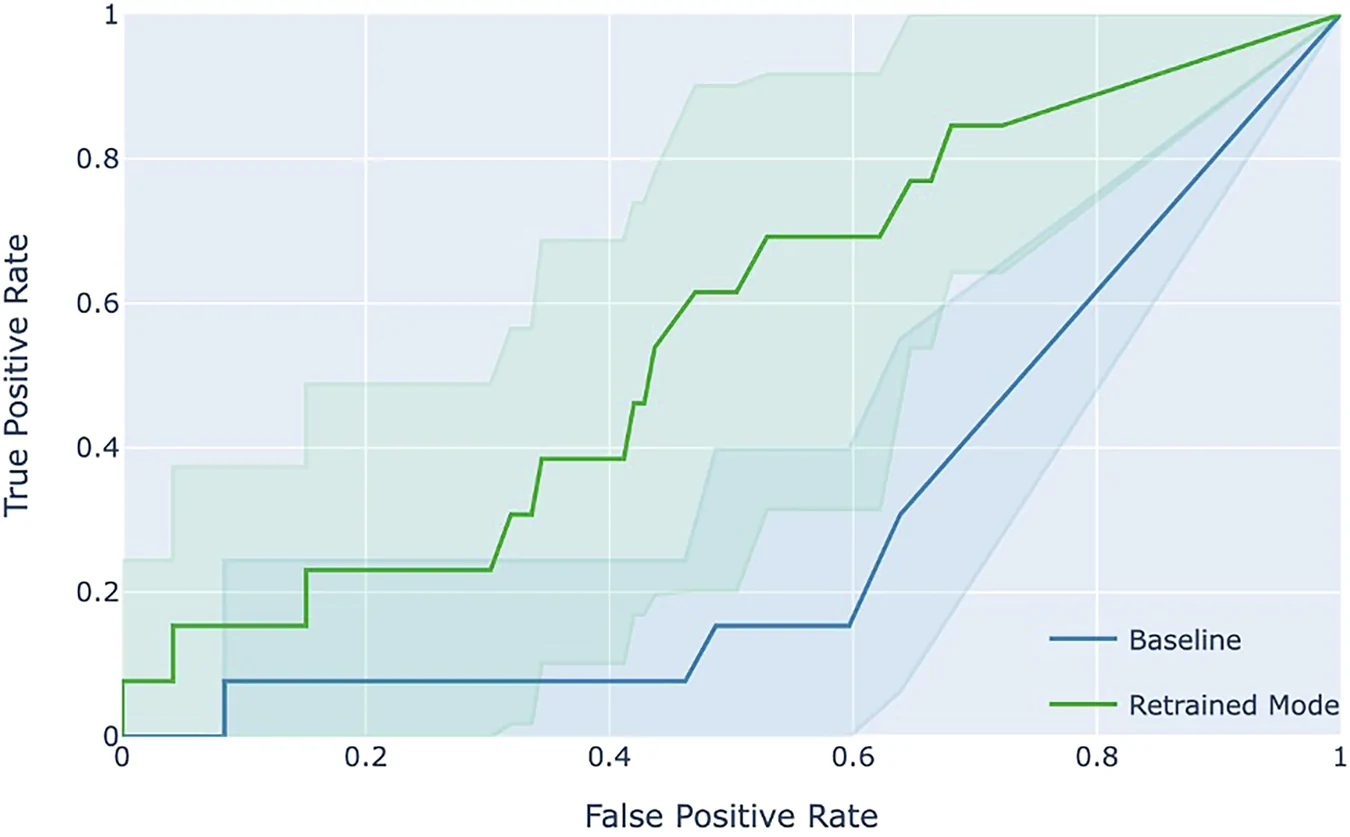
ROC curve for baseline and retrained models, for rib fracture detection. Note: The blue line represents the ROC curve for the baseline model, and the green line shows the ROC curve for the retrained model. The blue and green shaded areas represent 95% confidence intervals for the baseline and retrained model, respectively. Graph courtesy of Gleamer

**Fig. 5:**
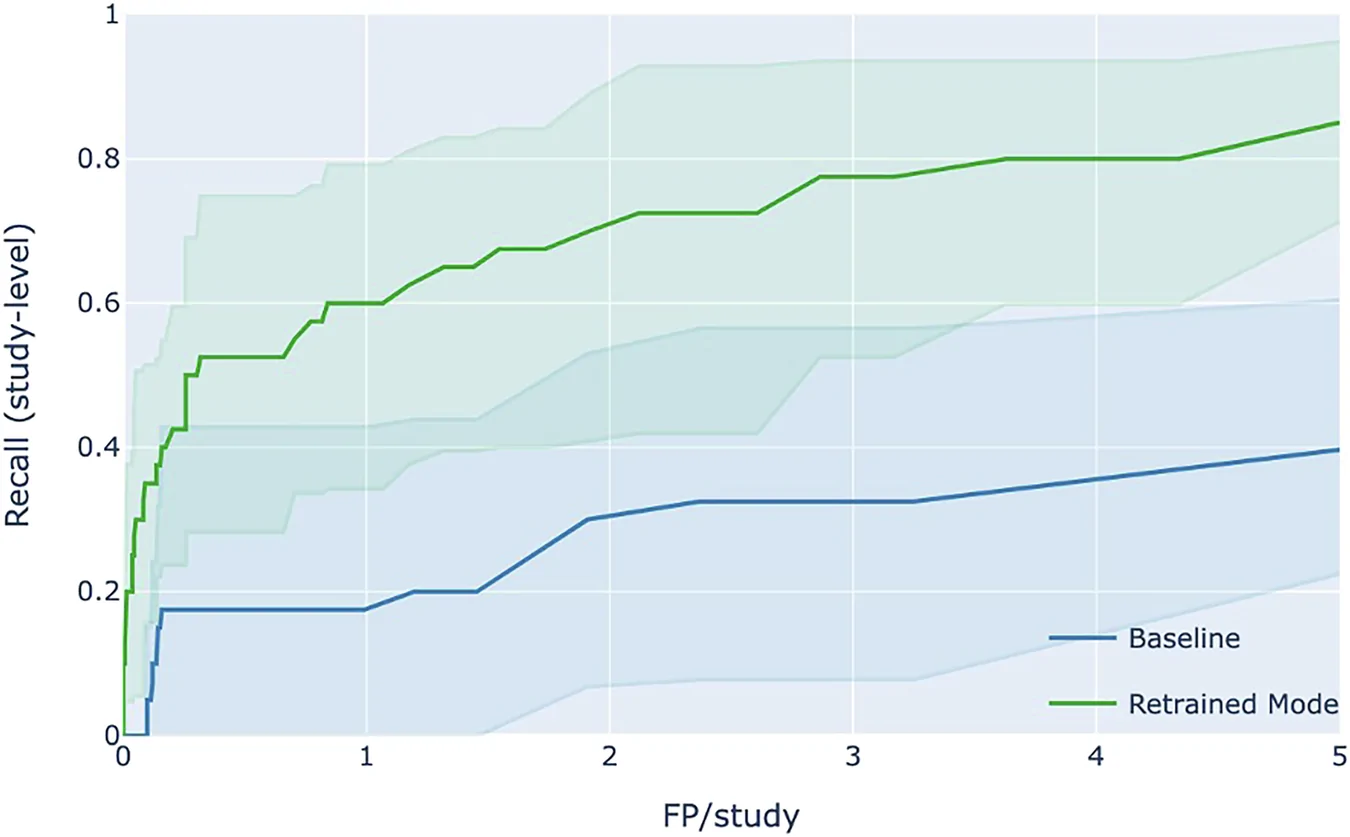
FROC curve for baseline and retrained models, for rib fracture detection. Note: The blue line represents the FROC curve for the baseline model, and the green line shows the FROC curve for the retrained model. The blue and green shaded areas represent 95% confidence intervals for the baseline and retrained model, respectively. Graph courtesy of Gleamer

Sub-analysis focused on rib fractures due to limited data for other fracture types. The baseline model had an AUC of 0.29 (95% CI: 0.19, 0.42), with a study-wise sensitivity at 8% (95% CI: 0%, 24%) and study-wise specificity at 76% (95% CI: 69%, 82%). Following retraining, the AUC increased to 0.56 (95% CI: 0.42, 0.73), with sensitivity improving to 23% (95%: 0%, 49%) and specificity remaining at 76% (95%: 71%, 83%) (Table 3). Figures 4 and 5 display the ROC and FROC curves, respectively, for the baseline and retrained models.

**Table 3:** Performance metrics for the baseline and trained models for the detection of rib fractures

	Baseline model	Retrained model
Study-wise sensitivity	8% (0%, 24%)	23% (0%, 49%)
Study-wise specificity	76% (69%, 82%)	76% (71%, 83%)
AUC	0.29 (0.19, 0.42)	0.56 (0.42, 0.73)

Sensitivity and specificity are calculated at an operating point of 0.5 false positives per study. Evaluation metrics are calculated at the study level, derived from the ROC curve. Values in parentheses are 95% confidence intervals, computed via bootstrapping

*AUC* area under the receiver operating characteristic curve

## Discussion

An independent initial evaluation of BoneView for the detection of inflicted fractures yielded poorer results compared with AI tools previously reported for accidental fracture detection [11–13, 15]. Therefore, BoneView was retrained to contribute to child SPA investigations. Preliminary retraining was performed using 329 annotated studies. After retraining, the model’s sensitivity and specificity improved by 8% and 6%, respectively. These results are promising given the small training set, suggesting further data could lead to clinically significant improvements in performance.

Our results, while preliminary, showed poorer performance compared to other AI tools for paediatric fracture detection [11–13, 15, 16]. However, this study is the first to develop an AI tool for the detection of inflicted fractures for multiple anatomical locations. Inflicted fractures are generally more subtle on radiographs compared to accidental fractures, suggesting that a larger dataset may be necessary to achieve good performance. Consequently, direct comparisons with existing literature are challenging. Our sub-analysis for rib fractures demonstrated a lower AUC, sensitivity, and specificity compared to overall model performance across all fracture types. However, significant improvements in AUC and sensitivity were observed following retraining, indicating rib fracture detection could become more reliable given further annotated data. Two previous studies have developed AI tools for detecting paediatric rib fractures, although not specifically in the context of SPA. Ghosh et al (a) [17] reported an AUC of 0.84. However, their study only included frontal chest views, whereas we included anteroposterior (AP) and oblique views, recommended as part of skeletal surveys [5]. For this reason, and because we expect to have a larger sample of rib fracture images (*n* ≈ 600) compared to Ghosh and colleagues (*n* = 486), we anticipate the fully retrained model to exceed the AUC achieved in their study. Ghosh et al (b) [18] developed an AI tool for rib fracture detection, achieving an accuracy of 63.46%, the lowest reported for any paediatric fracture detection AI tool in the literature. This corroborates our findings that the model’s diagnostic performance for rib fractures was notably lower than for other fracture types. A possible explanation is that chest radiographs contain more ‘noise’ due to the ribs overlapping with themselves, other bones (e.g., clavicle and spine), and organs (e.g., liver and heart), which can lead to more false positives [19]. Only one study has demonstrated the feasibility of developing an AI tool for detecting inflicted fractures. Tsai and Kleinman [20] trained a convolutional neural network to detect classic metaphyseal fractures of the distal tibia on skeletal surveys performed for SPA, achieving a sensitivity and specificity of 88% and 96%, respectively. Given our significantly larger dataset, our final results may surpass those of Tsai and Kleinman. While their normal cohort comprised 177 radiographs from 89 infants, and their fractured cohort consisted of 73 radiographs from 35 infants, our normal cohort is 2509 studies, and our abnormal cohort is 1227 studies (with approximately 20 radiographs per study). Theoretically, our larger dataset should yield a highly accurate algorithm. However, it is important to note that Tsai and Kleinman focused on fractures of the distal tibia, allowing the algorithm to specialise in that area. Training a CNN to detect fractures across the skeleton is more challenging.

There are several limitations to this work. The dataset was obtained from a single large tertiary centre, which may limit reproducibility in other settings (e.g., district general hospitals) due to variations in imaging and resources. Additionally, fracture patterns can vary by regional levels of social and economic deprivation, indicating our results may not apply to other regions [21, 22]. To mitigate this, future work should include data across multiple hospitals and regions. Another limitation lies in this study’s ground truth, which relied on radiologists’ opinions, whose accuracy is untestable without an external reference standard. To maximise the reliability of the ground truth, an experienced paediatric MSK radiologist with over 20 years of experience reviewed inconclusive or unavailable reports and validated all annotations. Moreover, following the 2017 RCR guidance, all skeletal surveys were double-reported by paediatric radiologists. While efforts were made for this research to closely align with real-world workflow, our pre- and post-training results are reflective of a single deep learning model inspired by BoneView and are not representative of the commercial software package, which is composed of several models. Finally, a general limitation of deep learning is its ‘black box’ nature. We could not fully understand the reasoning behind the predictions, as the algorithm does not explain them.

In conclusion, preliminary training of a deep learning model inspired by BoneView using a small number of annotated studies led to improvements in sensitivity and specificity (8% and 6%, respectively) for detecting inflicted fractures. This justifies further work to complete the annotation of the entire dataset, which we predict will further improve model accuracy. Sub-analysis by fracture type, age, displacement, and location will identify areas for further model improvement. Additionally, comparing model performance by initial and follow-up skeletal surveys will help determine whether a reliable AI tool could eliminate the need for follow-up surveys, saving time and stress for patients and families and costs for the NHS. In the future, multicentre collaboration should help to further improve the model.

## Supplementary information

Supplementary information

## Abbreviations

AI: Artificial intelligence
AUC: Area under the receiver operating curve
FROC: Free-response receiver operating characteristic
PACS: Picture archiving and communication system
ROC: Receiver operating characteristic
SPA: Suspected physical abuse
UK: United Kingdom
