# Supplementary material for: Developing an artificial intelligence tool for detecting fractures of child abuse: preliminary findings
Source: Eur Radiol. 2026 Apr 4;36(8):6274–81. doi: 10.1007/s00330-026-12513-8 (PMC13342033; doi:10.1007/s00330-026-12513-8)
Supplement: Supplementary file 1 — Supplementary information [file 330_2026_12513_MOESM1_ESM.pdf]

Developing an artificial intelligence tool for detecting fractures of child abuse: preliminary findings

ELECTRONIC SUPPLEMENTARY MATERIAL

Supplemental Figure 1. Workflow process for BoneView

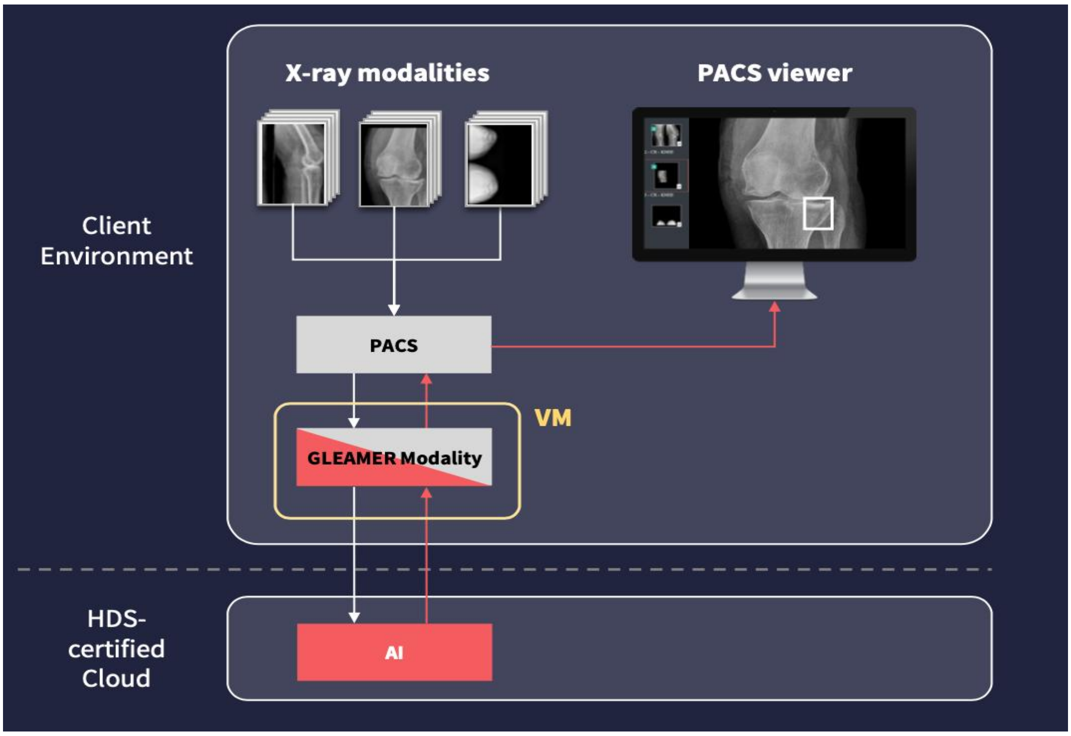

Note: Once installed, the software retrieves the images as they are pushed manually by a radiologist from PACS. Images are anonymised and sent to the AI algorithm on Gleamer's secure HDS cloud for analysis before being returned to PACS. The radiologist can then view the analysed image with/without the bounding box surrounding the abnormality, as well as the original unaltered image. Flowchart courtesy of Gleamer

Supplemental Table 1. Sensitivity and specificity for fracture detection at each anatomical region

**Site (skeletal survey examinations)**	**Sensitivity and Specificity based on anatomical site**						
		PACS report					
clavicle n=6		*positive*	*negative*	*sensitivity %*	*95% CI*	*specificity %*	*95% CI*
AI tool report	*positive*	2	3				
	negative	1	NC	66.7	16.1-96.7	NC	NC
		PACS report					
elbow n=1		*positive*	*negative*	*sensitivity %*	*95% CI*	*specificity %*	*95% CI*
AI tool report	*positive*	1	0				
	negative	0	NC	100	NC	NC	NC
		PACS report					

femur n=18		*positive*	*negative*	*sensitivity %*	*95% CI*	*specificity %*	*95% CI*
AI tool report	*positive*	8	1				
	negative	9	NC	47.1	28.4-77.9	NC	NC
		PACS report					
fibula n=4		*positive*	*negative*	*sensitivity %*	*95% CI*	*specificity %*	*95% CI*
AI tool report	*positive*	1	0				
	negative	3	NC	25	1.6-72.2	NC	NC
		PACS report					
foot n=3		*positive*	*negative*	*sensitivity %*	*95% CI*	*specificity %*	*95% CI*
AI tool report	*positive*	2	0				
	negative	1	NC	66.7	16.1-97.7	NC	NC
		PACS report					
hand n=3		*positive*	*negative*	*sensitivity %*	*95% CI*	*specificity %*	*95% CI*
AI tool report	*positive*	1	1				
	negative	1	NC	50	3.8-96.2	NC	NC
		PACS report					
humerus n=12		*positive*	*negative*	*sensitivity %*	*95% CI*	*specificity %*	*95% CI*
AI tool report	*positive*	8	2				
	negative	3	NC	72.7	43.5-92.4	NC	NC
		PACS report					
radius n=7		*positive*	*negative*	*sensitivity %*	*95% CI*	*specificity %*	*95% CI*
AI tool report	*positive*	3	3				
	negative	1	NC	75	27.8-98.4	NC	NC
		PACS report					
rib n=19		*positive*	*negative*	*sensitivity %*	*95% CI*	*specificity %*	*95% CI*
AI tool report	*positive*	2	2				
	negative	15	NC	11.8	2.1-32.1	NC	NC
		PACS report					
scapula n=1		*positive*	*negative*	*sensitivity %*	*95% CI*	*specificity %*	*95% CI*
AI tool report	*positive*	0	1				
	negative	0	NC	0	NC	NC	NC
		PACS report					
skull n=1		*positive*	*negative*	*sensitivity %*	*95% CI*	*specificity %*	*95% CI*
AI tool report	*positive*	0	0	0	NC	NC	NC

	negative	1	NC				
		PACS report					
tibia n=22		*positive*	*negative*	*sensitivity %*	*95% CI*	*specificity %*	*95% CI*
AI tool report	*positive*	8	6		26.9-73.1		
	negative	8	NC	50		NC	NC
		PACS report					
ulna n=10		*positive*	*negative*	*sensitivity %*	*95% CI*	*specificity %*	*95% CI*
AI tool report	*positive*	5	5				
	negative	0	NC	100	NC	NC	NC
		PACS report					
vertebra n=5		*positive*	*negative*	*sensitivity %*	*95% CI*	*specificity %*	*95% CI*
AI tool report	*positive*	0	0				
	negative	5	NC	0	NC	NC	NC

Abbreviations: *AI* artificial intelligence, *CI* confidence interval, *PACS* picture archiving and communication system, *NC* not calculable

Supplemental Table 2: Sensitivity for acute and healing fractures

**Age of fracture in Skeletal survey**	**Sensitivity and specificity**						
		PACS report					
Acute n=18		*positive*	*negative*	*sensitivity %*	*95% CI*	*specificity %*	*95% CI*
AI tool report	*positive*	9	0		28.1-71.9		
	negative	9	NC	50		NC	NC
		PACS report					
Healing n=57		*positive*	*negative*	*sensitivity %*	*95% CI*	*specificity %*	*95% CI*
AI tool report	*positive*	27	0		34.7-60.2		
	negative	30	NC	47.4		NC	NC

Abbreviations: *AI* artificial intelligence, *CI* confidence interval, *PACS* picture archiving and communication system, *NC* not calculable

Supplemental Table 3. Sensitivity for fracture types

Type of fracture in Skeletal survey	Sensitivity and specificity						
		PACS report					
Shaft n=14		*positive*	*negative*	*sensitivity %*	*95% CI*	*specificity %*	*95% CI*
AI tool report	*positive*	12	0				
	negative	2	NC	85.7	62.1-97.5	NC	NC
		PACS report					
Metadiaphyseal n=8		*positive*	*negative*	*sensitivity %*	*95% CI*	*specificity %*	*95% CI*
AI tool report	*positive*	7	0				
	negative	1	NC	87.5	55.5-99.2	NC	NC
		PACS report					
Supracondylar n=5		*positive*	*negative*	*sensitivity %*	*95% CI*	*specificity %*	*95% CI*
AI tool report	*positive*	4	0	80	37.2-98.7		
	negative	1	NC			NC	NC
		PACS report					
CML n=28		*positive*	*negative*	*sensitivity %*	*95% CI*	*specificity %*	*95% CI*
AI tool report	*positive*	8	0				
	negative	20	NC	28.6	14.2-46.7	NC	NC

Abbreviations: *AI* artificial intelligence, *CI* confidence interval, *PACS* picture archiving and communication system, *NC* not calculable
